# Identification of pathogens for differential diagnosis of fever with jaundice in the Central African Republic: a retrospective assessment, 2008–2010

**DOI:** 10.1186/s12879-017-2840-8

**Published:** 2017-11-29

**Authors:** Christelle Luce Bobossi Gadia, Alexandre Manirakiza, Gaspard Tekpa, Xavier Konamna, Ulrich Vickos, Emmanuel Nakoune

**Affiliations:** 1 Institut Pasteur of Bangui, Virology Department, PO Box 923, Bangui, Central African Republic; 2 Institut Pasteur of Bangui, Epidemiology Service, PO Box 923, Bangui, Central African Republic; 3 Hôpital de l’Amitié, Ministry of Public Health, Population and AIDS Control, PO Box 883, Bangui, Central African Republic

**Keywords:** Febrile jaundice, Differential diagnosis, Central African Republic

## Abstract

**Background:**

Febrile jaundice results clinically in generalized yellow coloration of the teguments and mucous membranes due to excess plasma bilirubin, accompanied by fever. Two types are found: conjugated and unconjugated bilirubin jaundice. Jaundice is a sign in several diseases due to viruses (viral hepatitis and arbovirus), parasites (malaria) and bacteria (leptospirosis). In the Central African Republic (CAR), only yellow fever is included on the list of diseases for surveillance. The aim of this study was to identify the other pathogens that can cause febrile jaundice, for better management of patients.

**Methods:**

Between 2008 and 2010, 198 sera negative for yellow fever IgM were randomly selected from 2177 samples collected during yellow fever surveillance. Laboratory analyses targeted four groups of pathogens: hepatitis B, C, delta and E viruses; dengue, chikungunya, Zika, Crimean–Congo haemorrhagic fever, West Nile and Rift Valley arboviruses; malaria parasites; and bacteria (leptospirosis).

**Results:**

Overall, 30.9% sera were positive for hepatitis B, 20.2% for hepatitis E, 12.3% for hepatitis C and 8.2% for malaria. The majority of positive sera (40.4%) were from people aged 16–30 years. Co-infection with at least two of these pathogens was also found.

**Conclusion:**

These findings suggest that a systematic investigation should be undertaken of infectious agents that cause febrile jaundice in the CAR.

## Background

Febrile jaundice, a frequent symptom of certain infectious diseases, appears clinically as a generalized yellow coloration of the integuments and mucous membranes, due to excess plasma bilirubin, and is accompanied by fever >37.5 °C in the morning and 38 °C in the evening. It may be due secondarily to the release of a pyrogenic substance or of haemoglobin into the blood due to haemolysis or to cholestasis, with a decrease or halt in biliary secretion of intra- or extra-hepatic origin [1–4]. In low- and middle-income countries, predominantly in sub-Saharan Africa, febrile jaundice usually occurs in the presence of parasitic infections (malaria, toxoplasmosis, schistosomiasis), bacterial infections (typhoid, typhus, borreliosis, leptospirosis) or viral infections (hepatitis and Lassa, Marburg, Ebola, Crimean–Congo and Hantaan viral haemorrhagic fevers, cytomegalovirus, mumps, measles, rubella and Coxackievirus) and is sometimes present in sickle-cell disease [3–11]. These infections are major public health problems in the region, and understanding of their local epidemiology could indicate differential diagnoses [12].

For over a decade, the World Health Organization (WHO) has conducted epidemiological surveillance for yellow fever in countries such as the Central African Republic (CAR). Operationally, any case of febrile jaundice is considered a suspected case of yellow fever and must be tested for yellow fever antigen (immunoglobulin M, IgM) in a qualified national laboratory. Samples that are negative for IgM suggest the involvement of other pathogens. In the CAR, 3220 suspected cases of yellow fever were reported to the reference laboratory for haemorrhagic fever at the Institut Pasteur of Bangui between 2007 and 2012; of these, 55 were positive [13]. We designed an exploratory study to identify the pathogens in the samples that were negative for yellow fever.

## Methods

### Database and target population

Data from nationwide serosurveillance for yellow fever between 1 January 2008 and 31 December 2010 were analysed retrospectively. The data are in the Epi Info database (Centers for Diseases Control and Prevention, Atlanta (GA), United States of America), which also contains information on the samples and sociodemographic data on the donors. The patients who gave samples met the WHO standard definition of suspected cases of yellow fever, i.e. any person with acute onset of fever and jaundice appearing within 14 days of onset of the first symptoms [14]. The samples consisted of 1–5 mL of venous blood drawn into dry tubes and transported in refrigerated sample carriers at 4–8 °C to the national reference laboratory at the Institut Pasteur of Bangui. The transport was ensured by trained focal points at CAR regional health facilities. The data obtained for each patient were: age, sex, location, symptoms (fever and jaundice or any sign of bleeding), history of vaccination against yellow fever, date of onset of symptoms and dates of blood sampling and transport to the laboratory. Serum was separated from each patient’s blood sample within 24 h and tested for yellow fever virus-specific fever IgM by enzyme-linked immunosorbent assay (ELISA). Positive results with ELISA were confirmed by quantitative polymerase chain reaction (qPCR). The remaining serum samples were stored at −20 °C at the Institut Pasteur of Bangui.

### Selection of samples and identification of pathogens

As the purpose of this study was to differentiate yellow fever from other infections that present with similar features (acute fever and jaundice), only samples that were negative for yellow fever were selected. The codes of 2177 samples negative for yellow fever IgM were entered on an Excel spreadsheet from laboratory registers, and 198 samples were randomly selected for this study on the basis of the availability of testing reagents. The corresponding serum samples were identified in the storage freezer. Four groups of pathogens were targeted: hepatitis B, C, delta and E viruses; dengue, chikungunya, Zika, Crimean–Congo haemorrhagic fever, West Nile and Rift Valley arboviruses; malaria parasites; and bacteria (leptospirosis). The appropriate laboratory technique was used to identify each pathogen.

ELISA was used to screen for hepatitis, with a Murex™ Diasorin kit for hepatitis B virus (to detect hepatitis B surface antigen), antigen detection of hepatitis delta (HD) virus and a Monolisa™ HCV Ag-Ab ULTRA kit (Bio-Rad) for hepatitis C virus (HCV). Leptospira infection was diagnosed with the Panbio Leptospira IgM ELISA (Standard Diagnostics, Republic of Korea). The results were interpreted according to the manufacturers’ recommendations. Hepatitis E (HEV), dengue (DENV), chikungunya (CHIKV), Zika virus (ZIKV), Crimean–Congo haemorrhagic fever (CCHFV), West Nile virus (WNV) and Rift Valley (RVFV) infections were detected by qPCR [15–22]. The sequences of primers and probes used to identify these viruses are presented in Table 1.

**Table 1:** Primers and probes used to detect arbovirus and hepatitis E virus

Primers and probes	Nucleic acid sequences of primers and probes
qPCR Zika virus (ZIKV)	
ZIKV forward	5-nt9271AARTACACATACCARAACAAAgTggT9297–3’
ZIKV reverse	5′ nt9352-TCCRCTCCCYCTYTggTCTTg-9373 -3’
ZIKV probe	nt 9304-FAM-CTYAgACCAgCTgAAR-BBQ-9320
qPCR Dengue total (DENV 1–4 system)	
DENT forward	5′- AGGACYAGAGGTTAGAGGAGA - 3’
DENT reverse	5′- CGYTCTGTGCCTGGAWTGAT −3’
DENT probe	FAM-ACAGCATATTGACGCTGGGARAGACC-TAMRA
qPCR Dengue subtypes (DENV1, DENV2, DENV3)	
DEN1 forward	5′ ATACCYCCAACAGCAGGAATT - 3’
DEN1 reverse	5′ AGCATRAGGAGCATGGTCAC −3’
DEN1 probe	FAM-TTGGCTAGATGGRGCTCATTCAAGAAGAAT-TAMRA
DEN2 forward	5′ TGGACCGACAAAGACAGATTCTT 3’
DEN2 reverse	5′ CGYCCYTGCAGCATTCCAA 3’
DEN2 probe	FAM-CGCGAGAGAAACCGCGTGTCRACTGT-TAMRA
DEN3 forward	5′ AAGACGGGAAAACCGTCTATCAA 3’
DEN3 reverse	5′ TTGAGAATCTCTTCGCCAACTG 3’
DEN3 probe	FAM-ATGCTGAAACGCGTGAGAAACCGTGT-TAMRA
qPCR Chikungunya (CHIK)	
CHIK forward	5′- AAGCTYCGCGTCCTTTACCAAG - 3’
CHIK reverse	5′- CCAAATTGTCCYGGTCTTCCT −3’
CHIK probe	FAM-CCAATGTCYTCMGCCTGGACACCTTT-TAMRA
qPCR Rift Valley fever virus (RVFV)	
RVFV forward	5′- AAA ggA ACA ATg gAC TCT ggT CA - 3’
RVFV reverse	5′- CAC TTC TTA CTA CCA TTC CTC CAA T − 3’
RVFV probe	FAM-AAA gCT TTg ATA TCT CTC AgT gCC CCA A -TAMRA
qPCR West Nile virus (WNV)	
WNV3p forward	5′ Agg TTA gWg gAg ACC CCg T - 3’
WNV3p reverse	5′ ggT TgT gCA gAg CAg AAg ATC −3’
WNV3p probe	FAM-CGGTTCCGGCGGTGGTTTCT-TAMRA
Conventional PCR Crimean-Congo haemorrhagic fever virus (CCHFV)	
CCHFV forward	5′ – TGGACACCTTCACAAACTC - 3’
CCHFV reverse	5′ – GACAAATTCCCTGCACCA - 3’
qPCR Hepatitis E virus (HEV)	
HEV forward	5′ GCCCGGTCAGCCGTCTGG −3’
HEV reverse	5′-CTGAGAATCAACCCGGTCAC −3’
HEV probe	FAM-CGGTTCCGGCGGTGGTTTCT-TAMRA

Malaria was diagnosed with Standard Diagnostics Bioline Ag-Pf and Ag-pan (Standard Diagnostics, Ref 05FK60, Republic of Korea), which contains antibodies targeting both PfHRP2 and lactate dehydrogenase specific to *P. falciparum* and other *Plasmodium* species (*P. vivax, P. ovale* and *P. malariae*).

### Data analysis

Stata 11.0 software was used to calculate proportions of test results. The results were compared according to sociodemographic characteristics with the chi-2 test. Statistical significance was assessed at *P* < 0.05.

## Results

Evidence of infection with at least one pathogen was found in 49.0% of samples (*n* = 97). Males under 35 years of age were the most commonly infected, but the difference was not statistically significant (*P* = 0.567 and 0.118, respectively). Of the 131 samples, 66.2% were from Bangui, the capital of the CAR, and its surrounding area, Ombella M’Poko (Table 2).

**Table 2:** Distribution of positive results for pathogens in differential diagnosis of yellow fever according to sociodemographic characteristics in the CAR (2008–2010)

Characteristic		Any positive result		Negative result		*P*
		No.	%	No.	%	
Age (years)	< 15	35	52.2	32	47.8	0.567
	16–24	24	52.2	22	47.8	
	25–34	21	50.0	21	50.0	
	≥ 35	17	39.5	26	60.5	
Sex	Male	43	43.4	56	56.6	0.118
	Female	54	54.5	45	45.5	
Province of residence	Bangui	41	51.9	38	48.1	0.446
	Bambari	1	50.0	1	50.0	
	Basse Kotto	6	40.0	9	60.0	
	Haute Kotto	3	60.0	2	40.0	
	Kembe	2	33.3	4	66.7	
	Lobaye	0	0.0	7	100.0	
	Mbomou	3	75.0	1	25.0	
	Nana Mambere	1	25.0	3	75.0	
	Ombela-M’poko	27	51.9	25	48.1	
	Ouaka	1	100.0	0	0.0	
	Ouham	5	55.7	4	44.3	
	Ouham Pende	5	50.0	5	50.0	
	Sangha Mbaere	0	0.0	1	100.0	
	Vakaga	2	66.7	1	33.3	

The predominant pathogens were hepatitis B (51.5%; 50/97), E (33.0%; 32/97) and C viruses (20.6%; 20/97). Co-infections were frequent, at a rate of 25.8% (25/97). Analyses for other pathogens (leptospira, DENV, CHIK and Zika, CCHV, WNV and RVFV) yielded negative results. The results for 16 patients showed co-infection with hepatitis B, E and C viruses (Table 3).

**Table 3:** Pathogens identified in samples negative for yellow fever IgM, CAR, 2008–2010

Infectious agent	No. of samples tested^a^	No. positive (%)
HBsAg	162	32 (19.8)
HEV^b^	198	27 (13.6)
HCVAg	162	9 (5.6)
*P. falciparum*	198	4 (2.0)
Anti-HD^c^	50	17 (34.0)
HEV + HBsAg	162	6 (3.7)
HBsAg + HCVAg	162	4 (2.5)
HBsAg + *P. falciparum*	162	4 (2.5)
*P. falciparum* + HCVAg	162	3 (1.9)
HCVAg + HEV	162	2 (1.2)
HEV + *P. falciparum*	198	2 (1.0)
HBsAg + HEV + *P. falciparum*	162	2 (1.2)
HBsAg + HEV + HCVAg	162	1 (0.6)
HBSAg + *P. falciparum* + HCVAg	162	1 (0.6)
Other pathogens^d^	198	0 (0,0)

^a^After testing sera for leptospirosis; dengue arbovirus; chikungunya, Zika, Crimean–Congo haemorrhagic fever, West Nile and Rift Valley viruses; and *P. falciparum*, sufficient quantities of serum remained for testing only a further 162 samples

^b^Hepatitis E virus, tested with qPCR

^c^Hepatitis delta virus

^d^Leptospirosis; DENV; CHIK, ZIKV, CCHFV, WNV and RVFV

## Discussion

Almost half the samples from patients presenting with fever and jaundice contained at least one infectious agent. Hepatitis B, C and E viruses were the most common pathogens identified in our samples; these infections are endemic in the CAR [23, 24]. The prevalence of hepatitis B virus infection was higher than that found previously in the CAR by Komas et al., at 10.6% [25]; however, those authors collected samples from apparently healthy individuals, while we studied patients presenting with clinical symptoms.

HDV is a satellite of hepatitis B virus, and infection with this virus aggravates acute and chronic liver disease. A review of the literature shows strong variations in the prevalence of this virus among Africans seropositive for HbsAg, and the presence of this marker may constitute support for the argument that it plays a role in the development of HBsAg-associated liver diseases [26]. A study conducted in the CAR in 1989 showed that delta virus was the cause of fulminating hepatitis with severe jaundice in 124 cases [27]. In another study, however, it was much more common in patients with chronic liver disease [28].

Hepatitis E virus is endemic in central Africa, due mainly to poor environmental hygiene, a deteriorating health network and very poor epidemiological surveillance. Hepatitis E virus outbreaks were reported, for example, in 2002, 2004 and 2005 in Bangui [29, 30], and imported hepatitis E virus was documented in 2011, prompting implementation of the International Health Regulations (2005) in the CAR [31].

Severe malaria caused exclusively by *P. falciparum* in the CAR is also considered in people presenting with febrile jaundice, as this disease is endemic in the area. In severe malaria, jaundice is related to severe haemolysis, hepatocellular damage, drug toxicity or a combination [32–34]. Co-infections with these pathogens are alarming because of their potential synergy in hepatitis dysfunction. The co-existence of malaria and viral hepatitis in developing countries like the CAR may thus present a diagnostic dilemma and severe complications and delay treatment [35].

We did not identify most of the less prevalent infections, such as leptospirosis, dengue, chikungunya, Zika, Crimean–Congo haemorrhagic fever, West Nile or Rift Valley fever, despite the fact that Africa has become a hotspot for leptospirosis research by microbiologists working to differentiate the causal agents of yellow fever [36].

More than half (51.0%) the cases of febrile jaundice had none of the studied pathogens. This was to be expected, as other differential diagnoses of fever with jaundice include community-acquired sepsis, amoebic fever, acute cholecystitis and choledocholithiasis [37].

The main limitation of this study is the relatively small number of samples selected for testing, which may therefore not adequately represent the target population. Specifically, the proportion of people with HCV infection in this sample would not be representative of the overall proportion in the CAR, as febrile jaundice is present in only a subset of people with newly acquired infection. Another limitation is that only samples that were negative for yellow fever were tested. Some of the samples positive for yellow fever virus might also yield positive results for other infectious pathogens [38] that have life-threatening synergistic effects.

## Conclusions

This study shows that acute hepatitis virus infections are still an important problem in the CAR. It is therefore essential to scale up hepatitis B vaccination and improve sanitation and environmental hygiene. Moreover, clinicians should be sensitized to the diagnostic dilemma of patients presenting with febrile jaundice. This study provides data on the prevalence of these diseases and the possibility of epidemics. Nationwide surveillance for yellow fever should be complemented by a survey of hepatitis viruses and other emerging haemorrhagic infections.

## Abbreviations

CAR: Central African Republic
CCHFV: Crimean-Congo haemorrhagic fever virus
CHIK: Chikungunya
DEN: Dengue
DENT: Dengue total
DENV: Dengue virus
ELISA: Enzyme-linked immunosorbent assay
HBsAg: Hepatitis B surface antigen
HCV: Hepatitis C virus
HDV: Hepatitis delta virus
HEV: Hepatitis E virus
IgM: Immunoglobulin M
qPCR: Quantitative polymerase chain reaction
RVFV: Rift Valley fever virus
WHO: World Health Organization
WNV: West Nile virus
ZIKV: Zika virus
